# The Role of Positive Parenting and Maternal Well-Being of Low-Income Chilean Adolescent Mothers in Their Children’s Negativity

**DOI:** 10.3390/bs15091183

**Published:** 2025-08-30

**Authors:** Laura Léniz-Maturana, Rosa Vilaseca, Ruby Miranda-Osorio, Felipe Poblete-Valderrama, Patricio Baeza-Aguilar, Gustavo Pavez-Adasme, Viviana Rodas-Kürten

**Affiliations:** 1 Faculty of Law, Social Sciences and Education, Universidad Viña del Mar, Viña del Mar 2580022, Chile; ruby.miranda@uvm.cl; 2 Department of Cognition, Development and Educational Psychology, University of Barcelona, 08193 Barcelona, Spain; rosavilaseca@ub.edu; 3 Department of Sports Science and Physical Conditioning, Universidad Católica de la Santísima Concepción, Concepción 4330000, Chile; fpoblete@ucsc.cl; 4 Centro de Salud USS, Universidad San Sebastián, Valdivia 5091000, Chile; patricio.baeza@csuss.cl; 5 Dirección de Posgrados, Universidad Adventista de Chile, Chillán 3780000, Chile; gustavopavez@unach.cl; 6 Facultad de Salud, Universidad Santo Tomás, Valdivia 4780000, Chile; clararodasku@santotomas.cl

**Keywords:** adolescent mothers, positive parenting, maternal well-being, children’s socioemotional development, children’s negativity

## Abstract

This study analyzes the role of positive parenting, encompassing affection, responsiveness, encouragement, and teaching, on 79 children’s negativity aged 10 to 24 months during interactions with their adolescent mothers (aged 15 to 21). Parenting behaviors were evaluated using the Spanish version of the Parenting Interactions with Children: Checklist of Observations Linked to Outcomes (PICCOLO). Maternal well-being was assessed using the Spanish version of the Hospital Anxiety and Depression Scale (HADS). At the same time, the Ages and Stages Questionnaire-Socioemotional Development (ASQ-SE) measured mothers’ perceptions of their children’s socioemotional development. Children’s negativity was assessed using the Subscale of Negativity from the Early Head Start Research and Evaluation Project (EHSREP Negativity Subscale). Bivariate analysis revealed a significant association between maternal affection, responsiveness, encouragement, higher maternal anxiety, and maternal perceptions of their children’s socioemotional development with children’s negativity regarding anger, hostility, or dislike toward their mothers during interaction. Moreover, multivariate regression analysis showed that maternal affection, responsiveness, anxiety, and perceptions of children’s socioemotional development could predict negativity. The study underscores the significance of positive parenting, maternal well-being, and perceptions of children’s socioemotional development to prevent high levels of children’s negativity.

## 1. Introduction

One of the biggest challenges faced by Chilean adolescent mothers is overcoming poverty due to limited job opportunities and low education levels (Berthelon & Kruger, 2017). Moreover, adolescent motherhood in Chilean women does not always receive the required socioemotional support from their family, partner, or healthcare professionals, which critically undermines their sense of self-esteem and self-worth and their ability to function effectively within the maternal roles they are expected to assume (Mora-Guerrero et al., 2021). In the current Chilean context, one of the most detrimental aspects of adolescent motherhood is the growing inequality associated with it, which exacerbates disparities in opportunities and reinforces the intergenerational transmission of poverty, impacting both the mother and her children (Lavanderos et al., 2019). According to the Family Stress Model (FSM) (Conger et al., 2010), economic stress leads to parents feeling pressure and anguish from not being able to cover the basic needs of their children, influencing their psychological well-being and their parenting practices, which can become less sensitive and affectionate, affecting children’s socioemotional development (Masarik & Conger, 2017). In this sense, Carothers et al. (2006) noted that young mothers may experience emotional distress resulting from vulnerable living conditions, increasing the risk of social and emotional problems in their children, such as anger and hostility associated with children’s negativity (Brady-Smith et al., 1999).

Children’s negativity can arise from family circumstances marked by different factors such as socioeconomic disadvantages, maternal emotional distress, and parenting approaches (Choe, 2021). A Chilean study demonstrated that younger mothers tend to display less positive parenting behaviors compared to older mothers (Farkas et al., 2020), possibly attributed to a lower level of commitment of adolescent mothers, leading to less positive involvement with their children (Williams, 2020). Previous literature suggests that the lack of positive parenting interactions is associated with higher childhood externalizing problems and associated with children’s negativity (Cherry & Gerstein, 2023; Kullberg et al., 2023; Menashe-Grinberg & Atzaba-Poria, 2017; Parker & Benson, 2004; Trautmann-Villalba et al., 2006). In contrast, positive parenting during joint play activities has been shown to reduce children’s negativity (Menashe-Grinberg & Atzaba-Poria, 2017).

Furthermore, maternal distress, particularly anxiety and depression, can interfere with the emotional tone when interacting with their children (Nicol-Harper et al., 2007), impacting children’s self-regulation skills, especially in managing negative emotions (Planalp et al., 2022). In turn, maternal distress can increase the likelihood of perceiving the child as challenging or problematic (Santelices et al., 2021a, 2021b). In this sense, it would be interesting to investigate whether positive parenting, the emotional well-being of adolescent mothers, and maternal perceptions of their children’s socioemotional development would explain child negativity in the context of dyadic interaction. Analyzing this association is important because it is documented that greater maternal-child emotional availability and positive parenting are associated with less child negativity (Little & Carter, 2005).

To better understand this topic, it is crucial to investigate the concepts of positive parenting used in this research, maternal well-being, and their relationship to children’s socioemotional development and negative behavior.

### 1.1. Positive Parenting, Children’s Socioemotional Development, and Children’s Negativity

Traditionally, the concept of parenting has been understood as a variety of behaviors by primary caregivers to fulfill specific responsibilities to respond to their children’s needs through gestures and emotional expressions (Darling & Steinberg, 1993). This concept also considers parental attitudes that promote their children’s holistic well-being by caring for their health, feeding them, and providing a favorable environment to be pertinently stimulated (Eraso et al., 2006). In turn, it is well known that parental attitudes are associated with children’s expressions of negativity, such as yelling and screaming (Moed, 2024).

In this study, we will adopt the concept of “positive parenting” (hereafter referred to as “parenting”) based on Roggman et al. (2013b). This term characterizes the quality of parent-child interactions, emphasizing their role in promoting child development and supporting their learning. Dimensions of affection, responsiveness, encouragement, and teaching typify positive parenting. It has been employed in various research studies, whose samples have comprised both children with typical development (Rivero et al., 2021, 2023) and those with disabilities (Vilaseca et al., 2019a, 2019b, 2020, 2023; Innocenti et al., 2013). Additionally, this terminology, with its respective dimensions, has been applied across diverse cultural contexts, as documented in the studies by Roggman et al. (2013a) and Bayoğlu et al. (2013).

Affection and expression of warmth include physical proximity and affective verbal expression (Roggman et al., 2013b). Affection in mothers is associated with fewer problem behaviors in children (Zhang et al., 2020), higher social skills (Girard et al., 2017), and predicts lower externalizing problems (Reuben et al., 2016) and more prosocial behavior in children with better emotion regulation (Yavuz et al., 2022). In contrast, lower maternal warmth predicts children’s emotional maladjustment (Han et al., 2017), which may manifest as increased child negativity during interactions.

Maternal responsiveness refers to responding to the child’s signals, emotions, words, interests, and behaviors and is related to better children’s self-regulation (Bernier et al., 2010). Moreover, children who exhibit fewer externalizing problems tend to have mothers who show higher responsiveness. However, those children with behavioral problems often have mothers who are less responsive in their caregiving approach (Kochanska & Kim, 2013). The findings of Kochanska and Kim (2013) may be explained by the fact that it is well known that caregiver responsiveness is associated with less child negativity, with the child’s initiative to seek out the caregiver influencing both the maternal response and the child’s emotional expression (Dimachkie Nunnally et al., 2021).

Maternal encouragement, defined as fostering autonomy in children, is associated with increased independence, a higher sense of security, and better socioemotional development (Roggman et al., 2013b). Conversely, when maternal behavior leans toward extreme intrusiveness or overprotectiveness, it tends to correlate with higher levels of separation anxiety in children. (Zerrouk et al., 2024). Children’s anger is related to their mother’s negative parental behavior, making intrusiveness a risk factor in children’s emotion regulation (Feldman et al., 2011). Meanwhile, autonomy-supportive parenting is associated with better children’s social competencies (Su-Russell & Russell, 2021). Moreover, parental encouragement that supports children’s problem-solving and promotes emotional regulation and socially appropriate expressions of distress can reduce children’s negativity in terms of emotional reactivity and encourage more adaptive ways of coping with negative experiences (Howe & Zimmer-Gembeck, 2022).

Maternal teaching involves bringing cognitive and linguistic stimulation and promoting children’s participation in joint activities between mothers and children in conversations (Roggman et al., 2013b). According to Murray et al. (2016), maternal teaching is associated with better prosocial behavior in children. However, other studies have shown more consistently that parenting teachers are associated with better cognitive functions (Yue et al., 2019) and receptive and expressive communication in children (Nunes Cauduro et al., 2019). Despite this fact, in a study conducted by Shorer et al. (2025), it was found that if a caregiver who has difficulty regulating their emotions provides cognitive stimulation through playful interactions, it can contribute to reducing children’s negativity.

In sum, the positive parenting dimensions mentioned above are closely associated with children’s socioemotional development and with lower expressions of negativity in children. Negativity, often reflected in externalizing problems, negative affect, non-compliance, and dysregulation during parent-child interaction, tends to decrease when caregivers show emotional affection and responsiveness, encourage exploration to promote the child’s creativity, and show less intrusiveness that is available to stimulate their children (Menashe-Grinberg & Atzaba-Poria, 2017). Moreover, positive parenting, reflected in warmth, responsiveness, and playful interaction, which is associated with teaching parenting (Cerro & Vilaseca, 2018), is closely related to higher maternal perceptions of their children’s socioemotional development (Farkas & Rodríguez, 2017).

### 1.2. Maternal Well-Being, Children’s Socioemotional Development, and Children’s Negativity

It is well known that there are some adverse conditions during motherhood, such as the lack of social and family support, which contribute to the increase in emotional problems that can make them more likely to experience higher anxiety and depression (Gao et al., 2023). Furthermore, anxiety and depression can have a significant impact on children’s socioemotional development, especially when there are sociodemographic risk factors in economic and social terms (Madigan et al., 2018). Therefore, the low socioeconomic status of Chilean adolescent mothers (Berthelon & Kruger, 2017) and the lack of social support (Mora-Guerrero et al., 2021) could explain why, in some cases, a greater prevalence of higher maternal emotional distress is observed in adolescent than in adult mothers (Sangsawang & Sangsawang, 2023).

On the one hand, maternal anxiety from the prenatal period has a significant impact on a child’s socioemotional development in terms of less prosocial and problem behavior (Loomans et al., 2011). However, anxiety after the birth of the child may also have implications for the child’s negativity (Brooker et al., 2015). Specifically, postnatal maternal anxiety influences a variety of socioemotional outcomes, including a child’s negative affectivity and externalizing and internalizing dysregulation (Behrendt et al., 2020). According to Van Lieshout et al. (2020), the rate of anxiety is higher in adolescents who are mothers than in adult mothers or in adolescents who are not mothers, which can have adverse effects on the well-being of their children.

On the other hand, it is also known that maternal depression affects a child’s socioemotional development (Feldman & Eidelman, 2009) since it can negatively affect the mother–child interaction, which, in turn, might influence the child’s behavior problems (Edwards & Hans, 2015). In addition, it has been found that for mothers who are in a situation of social and economic vulnerability, depression has an impact on socioemotional development in the first five years of their children’s lives (Harris & Santos, 2020). Given that depression can be a chronic problem and the symptoms can be severe, it is essential to understand the implications for motherhood because mothers with high depression tend to report worse externalizing outcomes and social competence for their children (Morales et al., 2023). Given that measuring children’s development by parents can be advantageous because they observe their children in different situations for longer than external observers (Rothbart & Bates, 2007), it is important to understand how maternal anxiety and depression of adolescent mothers impact their maternal perceptions of their children’s socioemotional development, as indicated by Léniz-Maturana et al. (2022). The importance of this topic is underscored by the fact that the family is the first context in which children socialize and develop emotional behaviors, giving parents a significant influence on their children’s socioemotional development. Children’s socioemotional development problems may manifest in negative parent-child interaction (Santelices et al., 2021b).

It should be noted that younger maternal age and increases in maternal distress are related to higher mothers’ negative parenting behaviors (McFadden & Tamis-Lemonda, 2013). Therefore, it is essential to understand how parenting in adolescent mothers can explain their children’s negative behavior, taking into account their maternal well-being state and their maternal perceptions of their children’s socioemotional development; due to mothers with emotional distress, it can be challenging to assess the behavior of their children (Santelices et al., 2021a, 2021b).

Following on from the literature described in the introduction, the aims of the current study are (a) to examine the relation between a mother’s positive parenting (i.e., affection, responsiveness, encouragement, and teaching), maternal well-being (i.e., anxiety and depression), and maternal perceptions of their children’s socioemotional development with their children’s negativity, and (b) to describe the effects of parenting factors in terms of a mother’s positive parenting and maternal well-being with maternal perceptions of their children’s socioemotional development on their children’s negativity. Based on the background reviewed above, we propose the following research questions: How are mothers’ positive parenting, maternal well-being, and maternal perceptions of their children’s socioemotional development related to their children’s negativity? Moreover, do mothers’ positive parenting, maternal well-being, and maternal perceptions of their children’s socioemotional development impact their children’s negativity?

## 2. Materials and Methods

### 2.1. Participants

The study involved a total of 79 participants, comprising 42 boys (53%) and 37 girls (47%) who were all 24 months or younger (M = 15.5 months, SD = 4.2). The mothers of these children were between 15 and 21 years old (M = 19.1, SD = 1.7 years). To be eligible for the study, the following criteria were considered: (a) mothers needed to have experienced pregnancy at the age of 19 or younger, (b) the children had to be typically developing and aged from 10 to 24 months, and (c) low-income dyads were defined as households with a monthly income equal to or less than USD 407.17 for mid-low income and USD 196.19 for low-income, following the criteria established by the Chilean Association of Market Researchers in 2023 (AIM, 2023). Table 1 shows the characteristics of children and their mothers.

### 2.2. Instruments

A brief sociodemographic questionnaire was used to record sociodemographic variables related to the child (date of birth, gender, sibling, and attendance to preschool), the mother’s (age, educational level, job status, and if she was supported by the child’s grandparents, either during her work or study sessions, in her caregiving roles, which included feeding, toileting, and putting the child to sleep), and details about the living conditions of the mothers with their children (whether they reside with their families—grandparents and father—and monthly family income).

The Spanish version of the Parenting Interactions with Children: Checklist of Observations Linked to Outcomes (PICCOLO) (Vilaseca et al., 2019b, 2021) was employed to assess the parenting behaviors of adolescent mothers. Initially developed in the United States (Roggman et al., 2013b), published and distributed by Paul H. Brookes Publishing Co. Baltimore, MD, USA it is a well-established and validated measurement tool consisting of 29 items designed to evaluate parent-child interactions within the context of children aged between 10 and 47 months. These 29 items reflect parental behaviors associated with children’s developmental outcomes. They are rated based on their frequency, with scores ranging from 0 (absent, not observed), 1 (rare, minor, or emerging), to 2 (clear, definitive, strong, and frequent). The items are categorized into four domains: affection, responsiveness, encouragement, and teaching. PICCOLO generates individual scores for each dimension, ranging from 0 to 14 (and 0 to 16 for the teaching dimension), and an overall total score ranging from 0 to 58. The original PICCOLO has demonstrated good reliability, with a Cronbach’s Alpha coefficient of 0.91 for the total instrument (0.78 for affection, 0.75 for responsiveness, 0.77 for encouragement, and 0.80 for teaching). It also exhibits strong construct and predictive validity (Roggman et al., 2013b). In the Spanish version of the PICCOLO (Vilaseca et al., 2019b), intraclass correlation coefficients (ICC) ranged from 0.69 for the responsiveness domain to 0.85 for the total score. All domain and total scores demonstrated satisfactory Cronbach’s alpha coefficients (0.65 for affection, 0.75 for responsiveness, 0.76 for encouragement, 0.72 for teaching, and 0.88 for the total score). For the sample in this study (*n* = 79), the reliability (Cronbach α) for the total instrument was 0.87 (0.64 for affection, 0.70 for responsiveness, 0.69 for encouragement, and 0.62 for teaching). Inter-rater reliability was calculated based on 20% of the observations, yielding an agreement estimate of 0.85 for the total scores used in this study.

The Hospital Anxiety and Depression Scale (HADS), as adapted into Spanish by Caro and Ibáñez (1992) from the original instrument (Snaith, 2003) available through GL Assessment (London, United Kingdom), is a self-administered questionnaire consisting of 14 items. Respondents rate these items using a four-point Likert scale, assigning scores ranging from 0 to 3. HADS was employed in this study to assess anxiety and depression among participating mothers. Of the 14 items, seven correspond to depression, and the remaining seven correspond to anxiety. The scores for each item, depression and anxiety, are summed up to produce a total score for each dimension. This scale yields a total score ranging from 0 to 21 for each dimension, with higher scores indicating higher anxiety or depression (Zigmond & Snaith, 1983). Previous research has consistently demonstrated the reliability of the HADS. For anxiety, Cronbach’s alpha values have ranged from 0.68 to 0.93, with a mean of 0.83, while for depression, they have varied from 0.67 to 0.90, with a mean of 0.82 (Bjelland et al., 2002). Factorial analysis has consistently revealed a distinct two-factor structure in the Spanish version for all demographic groups, affirming the tool’s internal consistency and reliability (Quintana et al., 2003). The questionnaire’s validity has demonstrated acceptable to highly acceptable correlations with related constructs (Terol-Cantero et al., 2015). In our study, Cronbach’s alpha coefficient was notably high for anxiety (α = 0.83) but moderate for depression (α = 0.62).

Social-emotional child development was assessed by adolescent mothers using the Ages and Stages Questionnaire: Social-Emotional (ASQ-SE) (Squires et al., 2002). This tool is published and distributed by Paul H. Brookes Publishing Company (Baltimore, MD, USA). It is a questionnaire used to evaluate children’s social-emotional development (i.e., self-regulation, adaptive functioning, affect, social-communication, and interaction) from 1 to 72 months, and it is divided into nine age ranges. The ASQ-SE questions are rated on a three-point scale indicating if the child performs a behavior “often or always” (0), “sometimes” (5), or “never or rarely” (10). An additional check box allows respondents to indicate whether the behavior is of concern to the parents; a checked concern scores five additional points. The total score is calculated by summing their responses, with lower scores signifying better social-emotional development in children. Previous evaluations of this tool have consistently shown strong internal consistency, with Cronbach’s alpha values ranging from 0.71 to 0.90 and an overall value of 0.84 (Squires et al., 2002). In our study, the reliability was found to be α = 0.73.

The Subscale of Child Negativity from the Early Head Start Research and Evaluation Project: Child-Parent Interaction Rating Scales for the Three-Bag Assessment (EHSREP Negativity Subscale; Brady-Smith et al., 1999; Administration for Children and Families, DHHS Office of Head Start Maryland Washington DC, USA) was used to assess the negativity of children born to adolescent mothers, which is defined as the degree to which the child shows anger, hostility, or dislike toward the parent while they are interacting. The item is scored on a 7-point Likert scale from 1 (very low negativity) to 7 (very high negativity). In the current study, 25% of mother–child interactions were coded by two trained observers. The inter-rater reliability agreement was 0.80. In this sample, the child negativity score was from 1 to 6 (Mdn = 2.0, IQR = 1.0).

### 2.3. Procedure

Fifty health centers, five hospitals, and 43 preschool centers were contacted, in addition to the National Service for Children in the provinces of Arauco, Biobío, and Concepción, which belong to the Biobío Region, Chile. Of all the institutions contacted, seven health centers, one hospital, four preschool centers, and one residential home for adolescent mothers authorized the study. Subsequently, the institutions were asked to collaborate in obtaining personal data on the families, such as dates of birth, contact numbers, and home addresses. Once we contacted the families, we visited the mothers who agreed to participate in their homes. On the one hand, mothers aged 18 or older signed an informed consent form. On the other hand, mothers below 18 had to complete informed assent forms, and their parents had to complete an informed consent form. At that time, they were provided with information regarding the confidentiality and voluntary nature of their participation, along with the mothers’ right to withdraw from the study without consequences, to alleviate any pressure from the extensive data collection. The mothers received no compensation for their participation. At the visit, the mothers completed sociodemographic questionnaires and the Spanish version of HADS (Caro & Ibáñez, 1992). They were given a brief explanation of how to complete the documents, and doubts were clarified. Completing both questionnaires took about 20 min. Afterward, the Spanish version of the ASQ-SE (Squires et al., 2002) was applied to evaluate the habitual behavior of their children in 15 min. Finally, video recordings were made of the interactions between adolescent mothers and their children during a play session of approximately 10 min. They were instructed to interact and play in a usual manner. A selection of toys was provided to facilitate the interaction. This choice of toys was based on the “three bags” strategy, which consists of presenting parents with different types of toys or materials so that they have a variety of options for interacting with their children during play, which encourages spontaneity and adapts to the child’s individual preferences and interests (Fuligni & Brooks-Gunn, 2013). Given the context of high family poverty and limited resources for mothers, this strategy was implemented to provide play options and materials that would allow for meaningful interaction between mothers and their children. The PICCOLO (Roggman et al., 2013b) and the Early Head Start Research and Evaluation Project’s Child Negativity Subscale (Brady-Smith et al., 1999) were used to analyze and score the recorded interactions between mothers and children. The study was approved by the Ethics Committee of the Health Service of Concepción (protocol code 19-03-05, 30 May 2019) and the Health Service of Biobío (protocol code 82, 26 April 2019), following the Declaration of Helsinki (1964 and subsequent updates) and the Guidelines for Good Clinical Practice (GCP).

### 2.4. Data Analysis

The first aim of this study was to explore the association between a mother’s positive parenting, maternal well-being, maternal perceptions of their children’s socioemotional development, and their children’s negativity. Non-parametric tests were conducted using Kendall’s τ coefficient to analyze the relationships between these variables, as it is suitable for ordinal variables (Chen et al., 2022), such as the children’s negativity measured by the EHSREP Negativity Subscale (Brady-Smith et al., 1999).

Variables that were included in the previous bivariate analyses were calculated for a multiple linear regression model to predict the impact of PICCOLO (Roggman et al., 2013b), HADS (Zigmond & Snaith, 1983), and ASQ-SE (Squires et al., 2002) scores in the EHSREP Negativity Subscale (Brady-Smith et al., 1999). Thus, the regression model to predict the children’s negativity included seven potential factors: (1) mother’s affection; (2) mother’s responsiveness; (3) mother’s encouragement; (4) mother’s teaching; (5) mother’s anxiety; (6) mother’s depression; (7) maternal perceptions of their children’s socioemotional development. A stepwise selection criterion was used to select the variables for inclusion in the model. Specifically, a predictor was added to the model if its probability was F ≤ 0.05 and removed from the model if it was F > 0.10. Statistical analyses were carried out using IBM SPSS version 27 for Windows.

## 3. Results

Table 2 shows statistical descriptors that include mean, standard deviations, minimum, and maximum scores of the variables of this research, such as the domains of affection, responsiveness, encouragement, and teaching as measured by PICCOLO, maternal anxiety and depression assessed through HADS, children’s socioemotional development assessed using ASQ-SE (raw scores), and the children’s negativity that the Subscale of Child Negativity Early Head Start Research and Evaluation Project evaluated.

Research Question 1: How are mothers’ positive parenting, maternal well-being, and maternal perceptions of their children’s socioemotional development related to their children’s negativity?

Kendall’s correlation coefficients between mothers’ positive parenting (PICCOLO), maternal well-being (HADS), and maternal perceptions of their children’s socioemotional development (ASQ-SE), with their children’s negativity (Subscale of Child Negativity Early Head Start Research and Evaluation Project), were calculated (Table 3). Mother’s affection (r = −0.359, *p* < 0.001), mother’s responsiveness (r = −0.332, *p* < 0.001), and mother’s encouragement (r = −0.315, *p* < 0.001) were inversely correlated with children’s negativity. Meanwhile, maternal anxiety (r = 0.211, *p* = 0.020) and maternal perceptions of their children’s socioemotional development (r = 0.194, *p* = 0.029) were positively correlated with children’s negativity. These results mean that children’s negativity was higher in those children whose mothers demonstrated lower affection, responsiveness, and encouragement while they were interacting with them, and in those mothers who presented higher anxiety and declared higher socioemotional development problems in their children.

Research Question 2: Do mothers’ positive parenting, maternal well-being, and maternal perceptions of their children’s socioemotional development, impact their children’s negativity?

The results (Table 4) indicate that high children’s negativity can be explained by a linear combination of low mother’s affection (β = −0.27, *p* < 0.001; 95% CI = [−0.44, −0.24]) and responsiveness (β = −0.24, *p* < 0.001; 95% CI = [−0.95, −0.06]), high maternal anxiety (β = 0.41, *p* = 0.010; 95% CI = [0.02, 0.14]), and maternal perceptions of their children’s socioemotional development (β = 0.17, *p* = 0.001; 95% CI = [0.17, 0.64]). The multiple linear regression model accounted for 42.4% (adjusted R^2^ = 0.424) of the variance of the Subscale of Child Negativity Early Head Start Research and Evaluation Project scores (F (3, 75) = 25.52; *p* < 0.001).

## 4. Discussion

The purpose of the current study was to analyze the relationship between a mother’s positive parenting (i.e., affection, responsiveness, encouragement, and teaching), maternal well-being (i.e., anxiety and depression), and maternal perceptions of their children’s socioemotional development, exploring their relationship and effects on their children’s negativity. Our findings suggest that children were higher negative behavior when their mothers showed less affective, responsive, and encouraging and when their mothers were more anxious and declared higher socioemotional development problems in their children.

The results mentioned above align with a study by Khaleque (2017), which similarly demonstrated that mothers who displayed non-affectionate behaviors, particularly hostility, exhibited a significant association with children’s negative personality, including defensive independence and emotional unresponsiveness. Moreover, parenting characterized by warmth is associated with reduced children’s negative behaviors regarding anger (Kochanska et al., 2004). This finding is interesting because, as in adult mothers, maternal affection can contribute to children having fewer problematic behaviors (Zhang et al., 2020), better development of social skills (Girard et al., 2017), and a lower probability of presenting externalizing problems (Reuben et al., 2016).

The relationship between mothers’ affection and low childhood negativity could be explained by the fact that, according to Watkins-Lewis and Hamre (2012), children who have received emotional warmth and affection in their homes may feel more secure. Therefore, maternal affection can be considered a protective factor against childhood anxiety and stress since providing a safe environment that promotes their emotional well-being can reduce the probability of their children engaging in harmful behaviors (Lansford et al., 2019; Philbrook & Teti, 2016).

Regarding the association between maternal responsiveness and child negativity, our findings are consistent with those of Menashe-Grinberg and Atzaba-Poria (2017). The authors point out that mothers who sometimes respond to children’s needs during play interactions have children with lower negativity. Likewise, it has been found that children who have less responsive mothers show higher negativity (Howard et al., 2011). In contrast, high parental responsiveness is often associated with better emotional regulation and social skills in children (Zarra-Nezhad et al., 2014), which could lead to a lower likelihood of childhood negativity.

A possible explanation for the relationship between high maternal responsiveness and low negativity is that parents who are sensitive and responsive to their children’s emotional signals need to establish an environment of security and confidence that improves secure attachment (Harder et al., 2015; Tamis-LeMonda et al., 2013). In this sense, parental responsiveness can influence children’s better ability to interact with others (Guttentag et al., 2014), including their closest environment, which, in this case, are mothers.

In our findings, there was also an association between higher maternal encouragement and lower child negativity. This result is similar to other studies in which it has been found that those mothers who do not promote autonomy in their children, but, on the contrary, are intrusive, have children who demonstrate higher negativity (Ispa et al., 2004; Menashe-Grinberg & Atzaba-Poria, 2017). Lack of support for children’s autonomy and not encouraging them to explore independently can hurt emotional development (Cooper-Vince et al., 2014) since the child may feel frustrated or anxious, which may affect their ability to regulate emotions (Wood, 2006). Specifically, it has been found that children of mothers with a higher tendency to restrict child autonomy are more likely to be more insecure (Raby et al., 2015).

Unlike the domains of affection, responsiveness, and maternal encouragement noted above, there was no significant relationship between maternal teaching and the child’s negativity. As the introduction notes, the teaching domain is more consistently associated with better cognitive (Yue et al., 2019) and language development in children (Nunes Cauduro et al., 2019). A possible explanation for this is that in the PICCOLO instrument (Roggman et al., 2013b), the teaching domain includes cognitive stimulation, explanations, conversation, joint attention, and shared play. Therefore, it is reasonable that teaching has the most significant association with children’s cognitive and communication development. In previous studies in which PICCOLO has been used (Roggman et al., 2013b), the teaching domain is associated with the cognitive, expressive, and receptive language areas, both in families with children who have typical normative development, and those who have disabilities (Vilaseca et al., 2019a, 2019b, 2020, 2023; Rivero et al., 2023). Despite this, future studies with adolescent mothers must analyze the relationship between a mother’s teaching and cognitive and linguistic development, as this research did not examine these variables.

It should be noted concerning the parenting of adolescent mothers that previous studies have shown that they stimulate their children less and pay less attention to their needs when interacting with them (Crugnola et al., 2014) and, in addition, are less sensitive (Ierardi et al., 2022). The above is usually linked to the low educational and economic levels of adolescent mothers, which leads them to experience emotional discomfort, these being risk factors for children’s socioemotional development (Weiss & Leung, 2021) since they are usually stressful situations that are related to parenting conflicts, affecting the socioemotional development in children of adolescent mothers (Jahromi et al., 2023). Furthermore, the transitory process of motherhood when they are very young makes it difficult for them to care for their children and adds to the stress that poverty generates (Erfina et al., 2019); however, when they have positive interactions with their children, they show better socioemotional development (Williams, 2020).

Regarding the adolescent mothers’ well-being, it can be seen that the scores obtained on the Hospital Anxiety and Depression Scale (HADS) revealed that the average anxiety and depression among the mothers who participated in this study did not reach high levels. The average was within a low range since 7 points or less indicates the absence of symptoms associated with anxiety or depression disorders (Snaith, 2003). This finding is interesting because previous evidence has shown that adolescent mothers are more likely to present mental health problems (Dinwiddie et al., 2018), given that they do not have social support, affective, emotional, informative, or economic (Peter et al., 2016), but these aspects were not evaluated in this study.

Despite the low scores of anxiety and depression in adolescent mothers who were part of this sample, it was found that those who presented higher anxiety had children with a higher childhood negativity. This finding was expected since previous scientific evidence has shown that low anxiety contributes to mothers having a higher feeling of optimism and parental competence, reducing self-regulation problems and negative affectivity in children. (Van den Heuvel et al., 2015). However, it is curious that there was no relationship between maternal depression and childhood negativity. It is well known that on some occasions, this negativity is measured by the perception of the depressed mother and that maternal symptoms of depression are related to less sensitivity towards their children (Savage & Birch, 2017). Therefore, they could not perceive the actual socioemotional development of their children, whether there are lower or higher problems in their behavior. However, more research with a more extended sample of depressed and anxious adolescent mothers is required to delve deeper into this issue.

Despite this, a positive association existed between maternal perceptions of their children’s socioemotional development and children’s negativity. According to Mäntymaa et al. (2006), the perception that their children are problematic is associated with a less favorable quality of the dyadic interaction between mothers and children, with maternal emotional distress being an essential factor. Therefore, our results make sense if we take into account that maternal anxiety was related to the socioemotional development evaluated by adolescent mothers and also to childhood negativity measured during mother–child interaction. In turn, the variables of low anxiety and evaluation by adolescent mothers of children’s socioemotional development, together with high maternal affection and responsiveness, explained lower childhood negativity. This result was also expected since affection and responsiveness are associated with more secure attachment relationships with their children (Friedman & Boyle, 2008); however, when children feel insecure in the first years of life when interacting with their caregivers, they are more likely to have higher negativity.

Therefore, our results show that lower maternal anxiety, as well as higher affection, responsiveness, and maternal perceptions of their children’s socioemotional development, would explain a lower childhood negativity. As previously noted, depression did not appear to have a significant role in childhood negativity, nor did the parental dimension of teaching or encouragement. Regarding the lack of association between maternal depression and childhood negativity, there is existing evidence of results similar to ours, which have shown that, unlike depression, anxiety has a significant relationship with maternal-infant bonding on the socioemotional behavior of their child, but not depression (Davies et al., 2021).

About the dimension of encouragement and teaching measured with PICCOLO, a previous study of Chilean adolescent mothers carried out by Léniz-Maturana et al. (2023) demonstrated that the dimension of encouragement was more related to child problem-solving but not to social development. Regarding the teaching dimension, as has been said throughout this article, it is instead associated with the development of language and cognition (Rivero et al., 2023; Vilaseca et al., 2019a, 2020, 2023). Our results call for continued research on parenting in low-income adolescent mothers and their relationship with child well-being and development. The importance of this lies in the fact that our findings provide evidence of the relevance of parenting in adolescent mothers since scientific evidence has shown that the quality of interaction between young mothers and their children significantly influences social and emotional child development (Crugnola et al., 2014), as shown by the results of this research. Particularly, it stands out that the parental competencies that contribute to less childhood negativity are associated with affection and responsiveness, as well as with maternal emotional well-being, as has been shown in other research (Gülseven et al., 2018; Enelamah et al., 2024).

The current research contributes valuable insights to the existing knowledge on the relationship between maternal well-being and parenting interactions in adolescent mothers with their children. This study adds to the current literature on the association between these parental factors and children’s negativity when interacting with their adolescent mothers. However, it is essential to acknowledge certain limitations in the study that should be considered when interpreting the results.

Firstly, adolescent mothers who agreed to participate were confirmed by a nonprobabilistic sample, which could introduce bias, as it may not represent the broader population of adolescent mothers. Thus, the method employed to recruit adolescent mothers in this sample could have been influenced by their willingness to participate (Hoffman et al., 2006). Then, it is probable that adolescent mothers who took part were the ones with better mental health and the most informed about the importance of positive parenting on their children’s socioemotional development. Nevertheless, adolescent motherhood in Chile, as in other Latin American and Caribbean countries, is a source of growing concern because it primarily affects young women with limited resources and lower educational levels (UNFPA, 2020). School dropout rates and limited job opportunities hinder their ability to overcome poverty (Berthelon & Kruger, 2017). In this sense, Chile is no different from other Latin American countries, so the results obtained in this research can be considered important for understanding phenomena related to this subject, both in Chile and in other contexts with similar characteristics in Latin America and the Caribbean. Therefore, the results of this study highlight the importance of providing social support and mentoring to adolescent mothers. This can be achieved through interventions tailored to their emotional needs and those of their children, which can contribute to the prevention of behavioral problems. For this reason, both adolescent mothers and their children should be recognized as a priority population in the support systems of institutional networks and national programs in Chile and other Latin American contexts.

Another limitation is the sample size, which limits the representativeness of the sample and the generalizability of the results. Seventy-nine participants is relatively small, which may limit the generalizability of the findings. Despite this fact, a study on parenting and its association with negative child behavior also was comprised of a sample of fewer than 80 participants, offering valuable insights into parental factors that influence child behavior, highlighting the potential of these findings to inform interventions aimed at promoting positive socioemotional development and improving children’s well-being (Saranya et al., 2025). Moreover, post hoc analyses revealed statistical power higher than 0.95 for 79 subjects, indicating a predictive ability of R^2^ = 0.20 in models with five predictors and α = 0.05. Therefore, relevant conclusions on effects can be obtained despite the sample size. It should be noted that this exploratory study may contribute to explaining the effect of specific parenting factors, which can later be analyzed with larger samples of adolescent mothers.

The use of stepwise regression could also be considered a limitation, as it has been criticized for its tendency to overfit, especially in small samples (McNeish, 2015). However, its application in this study is justified as an exploratory and practical procedure to identify relevant predictors within a small group of participants (*n* = 79). Although these findings should be interpreted with caution and considered as a basis for future research with greater statistical power, stepwise linear regression has also been used in other cross-sectional studies that have measured the quality of interaction between parents and children, recognizing that as an exploratory study it can contribute to the development of theoretical models that explain the mechanism of the effects of specific factors that can subsequently be used in larger samples (St George et al., 2017; Vilaseca et al., 2019a).

Lastly, some internal consistencies of PICCOLO’s subscales were low, and it could interfere with the psychometric properties of some of the instruments. However, it is not the first time in a sample of children with typical development (Rivero et al., 2022). It should be noted that PICCOLO has been applied in families of diverse countries, and the instrument has demonstrated strong reliability and predictive validity for low-income dyads of mothers and children (Bayoğlu et al., 2013; Roggman et al., 2013a; Vilaseca et al., 2019b). Therefore, despite this limitation, it is important to consider that few observational instruments assess positive parenting in the Chilean context. Although the Positive Parenting Scale (Muzzio & Quinteros, 2014) may be an alternative, it is a questionnaire answered by parents, which, according to its authors, can lead to potential social desirability biases, suggesting that PICCOLO is a suitable alternative. According to some authors, such as Roggman et al. (2013a), direct observation of parental interactions allows for more detailed and contextualized information on parenting in everyday situations. Furthermore, PICCOLO distinguishes itself from other instruments in assessing positive caregiver behaviors, which is relevant for approaches focused on family strengths (Roggman et al., 2013a, 2013b; Vilaseca et al., 2019b). It is worth noting, on the one hand, that PICCOLO has been previously used with Chilean adolescent mothers (Léniz-Maturana et al., 2023) and culturally adapted to a Chilean sample (Farkas et al., 2016), which supports its applicability in this context. On the other hand, in the literature that has examined the influence of parental factors on problematic child behaviors, relatively low levels of internal consistency have been accepted when the instrument used has been adapted to the sociocultural context of the country in which the research is conducted (Strauss et al., 2024), as is the case in this study.

Future studies about adolescent mothers’ parenting behaviors and their association with children’s negativity should include recorded observations of mothers’ and children’s interactions years later. The importance of analyzing the possible continuity of these dyad interaction patterns lies in the fact that it is well-known that maternal competencies in the Chilean sample tend to be better in older mothers compared to younger ones and in mothers who show higher educational levels and socioeconomic status (Farkas et al., 2020). Although these variables were not included in this study, our research findings support the idea that parenting factors of adolescent mothers may explain their children’s negativity. More specifically, our results may contribute to determining how maternal affection, responsiveness, encouragement, anxiety, and maternal perceptions of their children’s socioemotional development are associated with their children’s negativity. Due to adolescent mothers being part of a group with higher social and economic risk, promoting interventions to improve parenting competencies may enhance their children’s well-being and reduce their negative emotions.

Another aspect to consider in future research would be to incorporate the assessment of children’s socioemotional development based on direct observations of mother–child interactions, as has been implemented in other Chilean studies (Farkas et al., 2020; Ramos et al., 2020). As suggested by Léniz-Maturana et al. (2022), in studies involving adolescent mothers, it is important to combine questionnaires with direct observations to gain a better understanding of the children’s socioemotional development to avoid underreporting or overreporting their experiences. However, the focus of this research was to explore the association between maternal perceptions of their children’s socioemotional development and the negativity exhibited in dyadic interactions. In line with findings from Li et al. (2019), a significant relationship was found between emotional difficulties reported by mothers and childhood negativity. In this regard, the current study contributes important evidence on the influence that maternal perceptions of their children’s socioemotional development—along with other parental factors—can have on their children’s expression of negativity. The inclusion of this perception is especially valuable, allowing contrasts between mothers’ reports and the behaviors expressed by their children.

## Figures and Tables

**Table 1:** Characteristics of children and their mothers.

	N	%
Child		
Preschool center		
Attends	28	35.4
Does not attend	51	64.6
Siblings		
Yes	11	13.9
No	68	86.1
Mother		
Educational level		
Dropped out of the school	11	13.9
High school Student	18	22.8
High school graduated	50	63.3
Job status		
Employed	15	19.0
Unemployed	64	81.0
Childcare support		
Yes	62	78.5
No	17	21.5
Living conditions of mothers with their children		
Relatives living with mother and child		
Mother, father, and children	6	7.6
Mother, grandparents, and children	56	70.9
Mother, grandparents, fathers, and children	17	21.5

**Table 2:** Descriptive data.

	Min.	Max.	M	SD
Mother’s affection (0–14)	3	14	9.91	2.51
Mother’s responsiveness (0–14)	2	14	10.03	2.54
Mother’s encouragement (0–14)	1	14	8.78	2.70
Mother’s teaching (0–16)	0	14	8.59	3.02
Maternal anxiety (0–21)	0	20	6.23	3.83
Maternal depression (0–21)	0	15	5.13	3.11
Children’s Socioemotional development (0–240)	0	145	36.58	22.58
Children’s Negativity (1–7)	1	6	1.98	1.05

Note: Raw values of the children’s socioemotional development.

**Table 3:** Kendall’s correlations between PICCOLO, HADS, ASQ-SE, and Subscale of Child Negativity Early Head Start Research and Evaluation Project scores.

Variables	2	3	4	5	6	7	8
1. Mother’s affection	0.328 **	0.577 **	0.109	0.072	0.018	0.016	−0.359 **
2. Mother’s responsiveness		0.358 **	0.344 **	0.020	0.001	−0.220 **	−0.332 **
3. Mother’s encouragement			0.315 **	0.058	−0.018	−0.014	−0.315 **
4. Mother’s teaching				0.061	−0.045	−0.025	−0.148
5. Maternal anxiety					0.426 **	0.047	0.211 *
6. Maternal depression						0.096	0.141
7. Children’s socioemotional development							0.194 *
8. Children’s negativity							

* *p* < 0.05, ** *p* < 0.001.

**Table 4:** Linear regression model on Subscale of Child Negativity Early Head Start Research and Evaluation Project score.

Variable	B	S.E.	β	*t*	*p*-Value
Intercept	3.35	0.44		7.65	<0.001
Mother’s affection	−0.11	0.05	−0.267	−2.49	<0.001
Mother’s responsiveness	−0.10	0.05	−0.235	−2.13	<0.001
Maternal anxiety	0.11	0.02	0.414	4.77	0.010
Children’s socioemotional development	0.23	0.10	0.213	2.32	0.001

B: regression coefficient; S.E.: standard error; β: standardized regression coefficient.

## Data Availability

Data are available upon request of the corresponding author.
